# Forces at play: exploring factors affecting the cancer metastasis

**DOI:** 10.3389/fimmu.2024.1274474

**Published:** 2024-02-01

**Authors:** Farooq Riaz, Jing Zhang, Fan Pan

**Affiliations:** ^1^Shenzhen Institute of Advanced Technology (SIAT), Chinese Academy of Sciences (CAS), Shenzhen, China; ^2^Department of Oncology, First Teaching Hospital of Tianjin University of Traditional Chinese Medicine, Tianjin, China; ^3^National Clinical Research Center for Chinese Medicine Acupuncture and Moxibustion, Tianjin, China

**Keywords:** metastasis, metastatic cascade, premetastatic niche, tumor microenvironment, hypoxia, extracellular matrix, circadian rhythm, gut-microbiota

## Abstract

Metastatic disease, a leading and lethal indication of deaths associated with tumors, results from the dissemination of metastatic tumor cells from the site of primary origin to a distant organ. Dispersion of metastatic cells during the development of tumors at distant organs leads to failure to comply with conventional treatments, ultimately instigating abrupt tissue homeostasis and organ failure. Increasing evidence indicates that the tumor microenvironment (TME) is a crucial factor in cancer progression and the process of metastatic tumor development at secondary sites. TME comprises several factors contributing to the initiation and progression of the metastatic cascade. Among these, various cell types in TME, such as mesenchymal stem cells (MSCs), lymphatic endothelial cells (LECs), cancer-associated fibroblasts (CAFs), myeloid-derived suppressor cells (MDSCs), T cells, and tumor-associated macrophages (TAMs), are significant players participating in cancer metastasis. Besides, various other factors, such as extracellular matrix (ECM), gut microbiota, circadian rhythm, and hypoxia, also shape the TME and impact the metastatic cascade. A thorough understanding of the functions of TME components in tumor progression and metastasis is necessary to discover new therapeutic strategies targeting the metastatic tumor cells and TME. Therefore, we reviewed these pivotal TME components and highlighted the background knowledge on how these cell types and disrupted components of TME influence the metastatic cascade and establish the premetastatic niche. This review will help researchers identify these altered components’ molecular patterns and design an optimized, targeted therapy to treat solid tumors and restrict metastatic cascade.

## Introduction

Metastasis, a key term delineating the progression and spread of malignancies to the distant and surrounding tissues and organs, is known for the majority of cancer-related morbidities and mortalities (1). In general, tumor cells evade the primary tumor site during the process of metastasis and ultimately disseminate to farther and distant sites through the circulatory system (2). A huge number of cancer patients develop advanced metastasis, which provokes the terminal stage of diseases and shows resistance to the currently available therapeutic remedies. These characteristics of metastasis make it a lethal hallmark of tumors (3). Formerly, metastasis was regarded as a progressive stage of the tumor, frequently happening during the tumor progression phase (4); nevertheless, previous observations verified the incidence of metastasis often at the early stage of oncogenesis. This concludes that metastasis can arise at earlier and later stages of tumorigenesis. However, the two stages signify diverse pathogenesis, e.g., cells contributing to the early metastasis carry truncal mutations, while cells causing late metastasis indicate subclonal mutations (5).

It has been implicated that malignant cells lead to the failure of vital organs and account for the overwhelming majority (up to 90%) of cancer-related deaths rather than primary cancers (6). Despite the fact that metastasis is the major reason for impaired tumor therapy and increased mortality, it has not been investigated well. Mounting evidence suggested that a huge amount of tumor cells is unleashed in the circulatory system in tumor patients; however, melanoma-related *in-vivo* studies indicated that the number of metastasized cells is <0.1% of tumors (7).

The dispersion of tumor cells detached from their primary tumor sites and their ultimate tendency to originate secondary tumors in distant organs requires a multistep, extremely inefficient, and complex but deadly approach which is termed as an invasion-metastasis cascade (8–10). This classical cascade of events implies a series of basic steps, including the invasion of primary cancer cells into adjacent tissues locally; hematogenous transit involving the intravasation of these tumor cells into the bloodstream with improved survival rate; extravasation and arrest of tumor cells into the distant tissues’ parenchyma through vascular walls; origination of micro-metastatic colonies into the tissue parenchyma; and the colonization involving the successive growth of microscopic colonies into apparent and clinically observable metastatic lesions (11). These events have assisted in rationalizing this complex series of biological processes, which are obligatory for the progression of a specific malignancy toward the explicit metastasis (11).

The oncogenic transformation of tumor cells relies on the interaction of several factors which regulate the perforation of a tight regulatory network and attain a compatible microenvironment that ultimately expedites the oncogenic transformations and metastasis (12). Nevertheless, a detailed pathogenetic mechanism revealing the primary tumor’s formation still lacks the biological understanding of metastatic diseases. Besides, several breakthroughs in unifying our understanding of tumor behavior and diverse types of metastases have emerged. It is evident that compared to primary tumor cells, metastasis-initiating cells frequently display very diverse phenotypic and transcriptomic features (13). These properties make metastatic tumors resistant to the currently available conventional remedies effective (14). During the last 2 decades, a wide range of emerging anti-oncogenic drugs considerably extended the patients’ survival rate, with a 5-year survival rate of <20% among cancer patients with stage IV tumors (15). Despite substantial efforts, a clear knowledge of the underlying mechanisms involving the metastatic process and the variation defining the abilities of various cancer cells that establish metastases are still elusive. Therefore, urgent attention should be paid to understanding the pathogenesis of metastasis, thereby advancing molecular therapies to target metastasis. In this chapter, we summarized the latest developments in understanding metastasis to provide a comprehensive review of the role of the tumor microenvironment (TME), including the cellular and extracellular TME, in affecting metastasis to help researchers identify effective anti-metastatic therapy.

## Cancer hallmarks in the metastatic cascade

**Figure 1:**
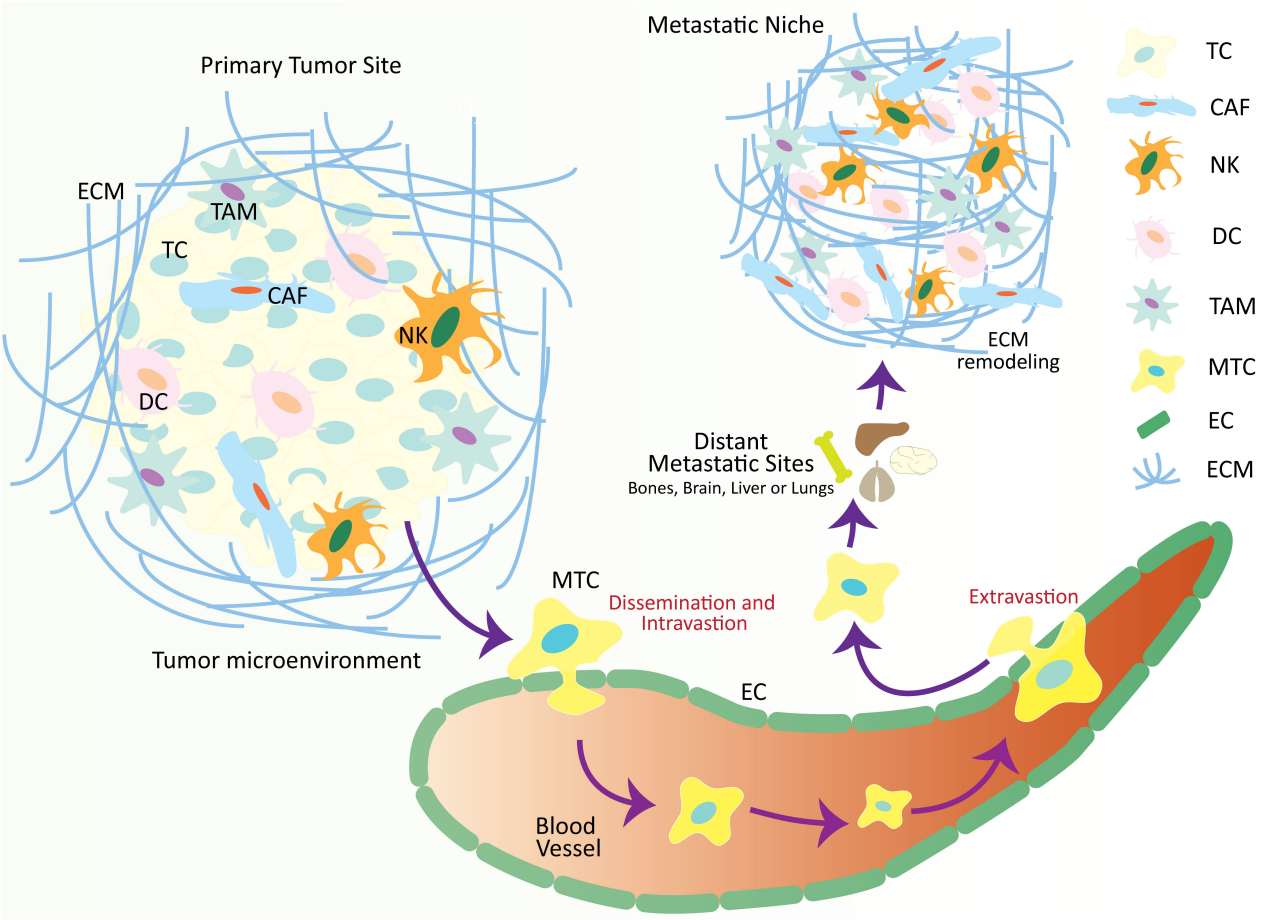
Schematic illustration over-viewing the physio-pathological processes of the metastatic cascade. The tumor microenvironment is comprised of numerous cellular and non-cellular factors that are involved in the process of tumor progression and metastasis. These factors initiate a series of cellular processes collectively called a metastatic cascade. During the metastatic cascade, metastatic tumor cells escape from their primary tumor site, enter the circulation, and translocate and thrive at a secondary tumor site in distant organs where a metastatic niche already exists, with favorable conditions for metastatic tumor development. TC, tumor cell; CAF, cancer-associated fibroblasts; NK, natural killer cell; DC, dendritic cell; TAM, tumor-associated macrophage; MTC, metastatic tumor cell; EC, epithelial cell; ECM, extracellular matrix.

Metastasis is characterized by the separation and local invasion of tumor cells from the primary site to the colonization and growth of metastatic cells at the secondary sites (11). The interaction of tumor cells with their adjacent stromal cells commences during the initial stage of cancer development. It continues with the onset of primary tumor growth, followed by invasion, intravasation, and colonization at the secondary site (11) (**Figure 1**). With time, it has become evident that TME contributes to tumor development at the secondary site by modulating the metastatic cascade (16). Investigations exhibit that TME is a complex entity that is comprised of a variety of components, including tumor-infiltrating immune cells, cancer-associated fibroblasts (CAFs), adipose cells, endothelial cells, extracellular matrix (ECM), and neuroendocrine cells (17). TME aggressively contributes to procuring cancer hallmarks, such as maintaining the growth rate of cancer cells, hindering cell mortality, enhancing the process of angiogenesis, avoiding immune destruction, triggering invasion and metastasis, and activating pro-tumor inflammation (18). These contributions of TME make it a striking therapeutic intervention.

## Cell types involved in the metastatic cascade

### Mesenchymal stem cells in the metastatic cascade

Mesenchymal stem cells (MSCs), also known as multipotent stromal cells, have the tremendous ability of self-renewal and the potential to differentiate themselves into other cell types, e.g., adipocytes, osteoblasts, and chondrocytes (19). These MSCs reside within the tumors where they significantly impact the TME development and function, thereby playing various roles at different stages of cancer progression. It is evident that MSCs influence the tumor cells by inducing their metastatic and invasive properties at the primary tumor site (20, 21), and aid in establishing a metastatic niche at the site of distant tumors (22). Meanwhile, MSCs also express their ability to differentiate and migrate into CAFs, especially in the tumor stroma, and induce the metastasis and growth of colon cancer by ameliorating the invasiveness, motility, and angiogenesis of tumor cells (23).

Multiple mechanisms have been reported by which MSCs exert their indispensable roles in promoting metastasis. It is believed that MSCs excrete TGF-β, which enhances the migratory and invasive capability of tumor cells (24). Besides TGF-β secretion, MSCs are the major source of exosome production (25). MSCs-derived exosomes facilitate metastasis by interacting with the tumor cells and influencing their migration and growth (26). A recent study utilizing the breast cancer cell line MCF7 determines that MSC-derived exosomes enhance the migration ability of tumor cells by inducing the target genes of the WNT signaling pathway, i.e., Axin2 and Dkk1, and increasing the level of β-catenin (27). Meanwhile, another study reported that the co-culture of MCF-7 cells with MSCs enhances the migration through the ER-SDF-1/CXCR4 axis (28). Besides, MSCs derived from bone marrow (BM) also influence the migratory ability of breast cancer cells by modulating the CXCR2 receptor (29). In lung cancer, MSCs are recognized to impact non-small cell lung cancer (NSCLC) cell metastasis. NSCLC leads to transcriptional alterations in MSCs, which enhances the factors involved in epithelial-mesenchymal transition (EMT) and metastasis, for instance, MMP-9. In short, MSCs activate the ABL tyrosine kinases during the progression of primary lung cancer, which is necessary for the increased expression of MSC-dependent MMP-9 (30).

Aryl hydrocarbon receptor (AhR) has been known to regulate the activity of immune cells (31) and tumor development through EMT modulation by transforming epithelial cells towards malignancy form (32). It was investigated that overexpression of AhR activates the EMT markers and enhances cell motility, invasion, and migration (32). Similarly, it was also found that overexpression of AhR via FICZ elevates the EMT markers and cell migration in triple-negative breast cancer (33). Also, the elevated level of AhR is associated with lymph node metastases (34) and/or poor prognosis in inflammatory breast and esophageal squamous cell carcinomas (ESCC) (34, 35). In the ESCC tumor microenvironment, modulation of AhR by using AhR activating ligand 3,3′-diindolylmethane decreased the levels of Vimentin and Slug along with reduction in the RhoA/ROCK1 signaling, which ultimately restricted COX2/PGE_2_ pathway, secretion of prostaglandin E2, EMT, cell migration and metastasis (35–37).

MSCs-derived exosomes carry numerous non-coding RNAs, mRNAs, and proteins (26). A study exploring the influence of MSC-derived exosomes on cancer metastasis suggested that MSC-derived exosomes collected from gastric tumors indulge the progression of gastric cancer through the MSC-derived exosomal miRNAs (38). Precisely, the elevated level of MSC-derived exosomal miR-221 during the pathogenesis of gastric cancer exerts a significant impact on the exacerbated lymphatic metastasis, tumor-node-metastasis stage, and local invasion (38, 39). Similarly, the exosomes secreted from MSC provoke several cellular processes, including EMT, and strengthen the invasion and migration of gastric tumor cells through the protein kinase B signaling pathway (40). Likewise, BM-derived MSCs also produce exosomes that stimulate multiple myeloma cell migration. These BM-MSC exosomes were suggested to embrace several cytokines, which ultimately participate in the expansion of numerous survival-related pathways, including Akt, p53, p38, and c-Jun N-terminal kinase (41). MSCs interact with and trigger M2 macrophages at distant metastatic sites to enhance the EMT and metastasis capability of tumor cells (42). Alternatively, MSCs in the TME directly modulate the levels of secretory CCL7 and TGF-β through KLF5/CXCL5 (43). Meanwhile, at the primary metastatic site, CAF-derived PAI-1 is an important factor promoting the metastasis cascade (44). Overall, it is evident that MSCs are the key players in metastatic cancer, and further comprehensive studies defining the interaction of tumor cells and MSCs are necessary to develop new anti-tumor drugs.

### Lymphatic endothelial cells in the metastatic cascade

The primary path of tumor dissemination is the lymphatic vessel (LV) located within the TME. Compared to normal blood vessels, LV is leaky (45). Increasingly studies have demonstrated the crucial role of lymphatic endothelial cells (LECs), which formulates the lining of LV, in the TME. It was revealed that LECs impact tumor progression and metastasis by significantly regulating the tumor cell-derived immune response within the TME (46, 47). The development of metastatic tumors at the distant site is triggered by the interaction of LECs and tumor cells (48). There are several factors that are secreted by the LECs to influence the impact of LECs on the various receptors in tumor cells (49). To recruit the tumor cells, LECs produce chemoattractants, i.e., CCL21 and SDF-1, which link to the chemokine receptors CCR7 and CXCR4 expressed in tumor cells (50). Meanwhile, distant tumors also produce multiple factors to facilitate the progression of the metastatic cascade, including recruitment, extravasation, and outgrowth, by conditioning the LECs. Among these factors, IL-6 is secreted by the tumor cells that activates the STAT3 in LECs and ultimately upregulates the VEGF expression (51), which is a necessary element for lymph angiogenesis (52). Moreover, this IL-6-dependent increase of VEGF expression in tumor cells is linked with the higher HIF-1α in LECs (52). Overall, it can be proposed that tumor-secreted factors directly influence the onset of lymphatic metastases. Further investigation regarding the interaction of LECs and tumor cells will be beneficial in treating cancer metastasis.

### Cancer-associated fibroblasts in the metastatic cascade

Cancer-associated fibroblasts (CAFs) are the primary cells that play an important role in TME (53). CAFs generally exert their roles in a variety of cellular processes, including cell stemness, cell proliferation cell differentiation, cell apoptosis, cell migration, and ECM remodeling. These cellular processes ultimately influence the biological behaviors of tumors, including tumorigenesis, tumor progression, proliferation, recurrence, immunity, tumor immunity, angiogenesis, energy metabolism, and metastasis (54, 55). Within solid tumors, CAFs consist of the most profound stromal components. These stromal components are biomarkers to easily distinguish CAFs from other cell subtypes. These markers include α-smooth muscle actin (α-SMA), platelet-derived growth factor receptor (PDGFRS), vimentin, fibroblast activation protein (FAP), integrin β1 (CD29), and podoplanin (56–58). It is evident that tumor cells activate fibroblasts in a series of events; for instance, during the early stages of tumor progression, tumor cells recruit fibroblasts, which undergo transformation towards the CAFs, and ultimately lead to performing their function in maintaining and reshaping the TME by inducing tissue repair; thereby play an anti-cancer role (58). It is noteworthy that activation of CAFs leads to the production of numerous signaling molecules which are used by tumor cells as growth factors for their proliferation and survival; thus, CAFs favor the growth of tumors and survival of cancer cells, and promote the recruitment and transformation of other cell types within the TME (58, 59). Meanwhile, CAFs produce MMPs, release collagen and fibronectin, and enhance VEGF expression to assist ECM remodeling (60–64). The CAFs-dependent ECM remodeling triggers the local invasion of tumor cells, and facilitates the onset of distant metastasis by providing essential survival conditions (65). To further assist the progression of metastatic tumors, several CAFs undergo reactivation at distant metastatic sites where they produce stromal components, e.g., periostin and tenascin, through various mechanisms that facilitate the colonization of tumor cells (66). It has been validated that a vigorous and aggressive cancer form can be observed if hypoxia-related factors, such as collapsed blood vessels and enhanced mechanical stress, combine with CAFs-triggered proliferative and pro-survival processes in tumor cells (67, 68).

A recent study revealed a new subtype of CAFs, namely, CAF-S1 in the microenvironment, which performs immunosuppressive functions by attracting and enhancing the growth, differentiation, activation, and survival of CD4^+^ CD25^+^ T cells (57). Additionally, increased CAF-S1 cells oblige the metastatic breast cancer to the distant organs through CDH11/osteoblast cadherin in patients with smaller (<2cm) primary tumors (69). Meanwhile, CAF-derived periostin endorses the tumor progression, metastasis, and cancer-stem cells (CSC) phenotype through the canonical Wnt/β-catenin axis in the head and neck squamous cell cancer (70). Another study pointed out that elevated YAP1 in CAFs increases the growth of tumor epithelial cells in prostate cancer and originates the secondary metastasis (71). CAF is also known to regulate the TGF-β pathway, which participates in the cancer progression through different processes, including tumor cell invasion, migration, proliferation, and ultimately metastasis (72). A study investigating hepatocellular carcinoma (HCC) progression showed that elevation of TGF-β1 increased the expression of connective tissue growth factor (CTGF). Moreover, the TGF-β receptor inhibitor enhanced the CTGF expression. It suppressed the proliferation of CAF=, thereby decreasing the tumor growth and indicating that targeting TGF-β can be an anti-metastatic therapy (73). Intriguingly, a recent study showed that CAFs dictate tumor cells to endorse migration by transferring mitochondria (74). Conversely, considering the role of IL1 signaling in shaping CAF heterogeneity, it was elucidated that pro-tumorigenic CAFs are characterized by IL1R1^hi^ expression, which is positively allied with immunosuppressive TME and metastasis (75). Overall, CAFs play a critical role in the metastatic cascade, and it is the need of the hour to understand the role of CAF-related activation pathways in metastatic tumors.

### Tumor-associated macrophages in the metastatic cascade

Macrophages exhibit diverse phenotypic and functional heterogeneity and play notable roles in regulating the immune response (76). Macrophages participating in the TME are referred as TAMs. These TAMs are widely spread among various tumors (77), representing miscellaneous functions in response to TME-related signals from cancer and stromal cells (78). TAMs play a significant role in drug resistance and promote tumor development, invasion, and metastasis (79). It was suggested that the plasticity of macrophages largely depends on their functional diversity, which is modulated by TME-associated molecules and signals (79). Comprehensive reports have demonstrated that the increased density and localization of TAMs, linked with the pathogenesis of several cancers and poor clinical outcomes, play a critical role in the TME (80).

Even though limited studies have focused on the role of TAMs in metastasis, a recent investigation highlighted that activation of TAMs produces proteolytic enzymes and soluble factors, including matrix metalloproteinases (MMPs), which directly execute substantial effects promoting metastasis (81). Similarly, M2 cells are one of the principal cells that initiate cancer development in both primary and secondary sites. M2 contributes to the process of metastasis by regulating the angiogenesis, breakdown, and deposition of the basement membrane, recruiting leukocytes, and suppressing the immune system (82, 83). M2 macrophages assist the migration of detached cancer cells or cancer stromal cells by destroying the matrix membrane of endothelial cells through the secretion of cathepsins, serine proteases, MMPs, and decomposition of numerous extracellular matrix components, including collagen (84, 85).

Despite the weak antigen-presenting capability, TAMs transform into M2-like phenotypes to support cancer development and metastasis (86). TAMs also secret numerous inflammatory cytokines, including IL-17 and IL-23, to induce tumor-derived inflammation, which sequentially propels tumor growth (87). Likewise, the upregulation of TAM-derived IL-6 strengthens the inflammatory responses and promotes HCC initiation and development (88). During the metastasis, TAMs and cancer cells develop a symbiotic relationship by releasing the epidermal growth factor (EGF) and colony-stimulating factor 1 (CSF-1), respectively. The interaction of these two factors leads to the migration of tumor cells (89).

TAMs, as a vital component of dissemination and metastasis, affect almost all stages of the metastatic cascade by interaction with tumor cells, ECM, and numerous other components of the immune system, particularly in lung tumors (90, 91). TAMs are a significant contributor to bone-metastatic prostate cancer. Tumor cells and TAMs interact to sustain androgen deprivation resistance during bone metastatic disease. TAMs exert androgen deprivation resistance through activation of activin A, which ultimately leads to elevated fibronectin (FN1), FN1-integrin alpha 5 (ITGA5), and tyrosine kinase Src (SRC) network in prostate cancer cells (92). Overall, TAMs exert protumoral functions, and TAMs-specific therapy may emerge as a striking therapeutic selection to prevent metastasis.

### Myeloid-derived suppressor cells in the metastatic cascade

Mounting proof demonstrates that myeloid-derived suppressor cells (MDSCs) are crucial to every metastasis stage. While the immune-suppressive activity of MDSCs plays a vital role in developing the metastatic niche (93, 94), these cells also use various strategies that promote metastases. Clinical evidence strongly suggests a potential role for myeloid-derived suppressor cells (MDSCs) in the metastatic process. In non-small cell lung cancer (NSCLC) patients, the presence of circulating CD14+HLA-DRlow monocytic MDSCs (M-MDSCs) was associated with extra-thoracic metastases (95). Similarly, an escalation in lymph node metastases among breast cancer patients correlated with an increase in indoleamine 2,3-dioxygenase (IDO)-expressing CD45+CD33+CD14−CD15− MDSCs within breast cancer tissue (96). Furthermore, a surge in circulating polymorphonuclear MDSCs (PMN-MDSCs) marked by CD11b+CD14-CD15+ and M-MDSCs in melanoma patients was linked to the initiation of metastases and reduced survival (97, 98).

The recruitment of MDSCs to the tumor site or the pre-metastatic microenvironment involves a complex interplay of chemokines and chemokine receptors. Existing research demonstrates that chemokines such as CXCL1, CXCL2, and CXCL5 play a pivotal role in attracting MDSCs to both the tumor site and the pre-metastatic microenvironment (99–101). The chemokine receptors CXCR2 and CXCR4 primarily recruit neutrophils, or PMN (polymorphonuclear)-MDSCs, to the premetastatic niches (102, 103). It has recently been demonstrated that, in contrast to naive neutrophils, bone marrow-derived neutrophils from early stages of cancer display higher levels of uncontrolled migration, higher levels of OXPHOS and glycolysis, and enhanced synthesis of ATP. These neutrophils also lack the immunosuppressive capability that is characteristic of MDSCs (104). Immunosuppressive bone marrow PMN-MDSCs from individuals with advanced cancer exhibit thorough variations in the cancer-specific neutrophils, suggesting the role of neutrophils in early tumor dissemination (105). Significantly, these cells demonstrated robust spontaneous migration, suggesting a potential for more effective movement toward non-inflammatory tissues compared to PMN-MDSCs or control neutrophils (93). It is conceivable that upon reaching their destination, these cells may undergo a transformation into PMN-MDSCs; however, this aspect has not been scientifically explored yet. PMN-MDSCs present in premetastatic niches may contribute to tumor cell escape through the inhibition of immune cells, induction of matrix remodeling, and stimulation of angiogenesis, all of which facilitate tumor cell engraftment (93). In a recent study, Li et al. reported an alternative regulatory mechanism of tumor metastasis, wherein neutrophils accumulated neutral lipids by suppressing the activity of adipose triglyceride lipase in a breast cancer model (106). The specific deletion of adipose triglyceride lipase in neutrophils was demonstrated to reduce metastasis in mice, highlighting the pivotal role of this enzyme in the process. It has also been established that lipid transfer from neutrophils to cancer cells promotes metastasis (106). According to the authors, these neutrophils exhibited a suppressive effect on NK cells, suggesting the potential identification of these cells as PMN-MDSCs.

Several recent investigations have shed light on the role of MDSCs in the EMT process. EMT is a phenomenon observed in certain tumor cells, wherein polarized epithelial cells undergo a transformation, losing their epithelial markers and adopting mesenchymal characteristics, facilitating their ability to spread, invade organs, and metastasize (107). In a notable study, Abastado et al. demonstrated the involvement of PMN-MDSCs in the RET transgenic mice model of spontaneous melanoma. PMN-MDSCs were attracted to the tumor site, and upon entering, they generated transforming growth factor-beta (TGF-β) and hepatocyte growth factor (HGF). These factors induced the initial melanoma cells to undergo EMT. Remarkably, reducing PMN-MDSCs in mice resulted in a decrease in EMT and subsequently reduced metastatic lesions (101). This research underscores the significant impact of PMN-MDSCs on promoting EMT and the subsequent metastatic process.

By augmenting the population of cancer stem cells or inducing a more stem-like state in cancer cells, myeloid-derived suppressor cells (MDSCs) actively promote cancer dissemination. The accumulation of Lin− CD45+ CD33+ MDSCs was correlated with diminished survival rates in both metastatic and non-metastatic ovarian cancer patients. Direct interaction between ovarian tumor cells and MDSCs resulted in the conversion of these cells into stem cells. The elevated levels of microRNA-101 in ovarian malignancies, targeting CtBP2—a co-repressor of stem cell genes—were identified as the mechanistic basis for this phenomenon. Moreover, when human ovarian tumor cells were co-cultured with MDSCs before inoculation into immunodeficient mice, there was an increased number of metastatic lesions in the liver and lungs and enhanced engraftment (108). In the context of pancreatic cancer, myeloid-derived suppressor cells of the monocytic subtype (M-MDSCs) directly stimulated the proliferation of aldehyde dehydrogenase-1+ (ALDH1) pancreatic cancer stem cells in a pancreatic cancer model. Similar outcomes were achieved using human CD14+ HLA-DR− M-MDSCs (109). This underscores the significant role of MDSCs in fostering the expansion of cancer stem cells, contributing to the progression and metastasis of cancer.

Although the role of MDSCs in modulating the immune response in various physiological and pathological settings, a substantial body of research supporting the pro-metastatic activity of MDSCs, a recent study has unveiled a surprising functional flexibility in MDSCs, revealing their potential to prevent metastasis in specific circumstances. In both non-metastatic and metastatic prostate and breast cancers, MDSCs were observed to accumulate in the pre-metastatic location of the lungs (110). In breast cancer, stress promotes the splenic MDSCs to form the pre-metastatic niche through the modulation of TAM/CXCL1 signaling (111). MDSCs derived from non-metastatic tumors exhibited elevated levels of thrombospondin-1 (TSP-1), a potent anti-angiogenic matrix protein, and demonstrated the ability to prevent metastasis. Prosaposin, a robust inducer of TSP-1, was identified as being released by non-metastatic tumors. Intriguingly, an amino acid peptide mimic of prosaposin was found to be sufficient to up-regulate TSP-1 in MDSCs *in vivo*, preventing tumor metastasis (110). On the other hand, the simultaneous implementation of ferroptosis induction and MDSC blockade restricts primary liver tumors and their metastases susceptible to immune checkpoint inhibition (112). This innovative approach challenges the conventional understanding of MDSCs in metastasis-promoting actions. Further research is imperative to corroborate the involvement of MDSCs in metastasis across diverse tumor models.

### T cells in the metastatic cascade

The quantity and quality of immune infiltrates in distinct metastases have been correlated with the progression of each individual lesion. Notably, regressing and stable metastases exhibited heightened infiltration of CD8+ and CD4+ T cells, along with T cell clonal expansion, in a patient with ovarian cancer undergoing chemotherapy (113). Conversely, concurrently growing metastatic tumors in the same individual displayed T-cell exclusion. Remarkably, both regressing and stable metastases demonstrated elevated expression levels of CXCL9, a chemokine implicated in T-cell trafficking (113). This observation underscores the interconnectedness of immune infiltration and treatment response, highlighting potential variations across metastatic lesions within a single patient. It is proposed that the efficacy of CD8+ T cell anti-tumor immunity plays a critical role in eradicating disseminated tumor cells (DTCs) in mouse melanoma and breast cancer models, as evidenced by promoting metastasis with T cell depletion (114, 115). Consequently, DTCs must withstand the assault from CD8+ T cells to develop into metastases successfully. Tumor cells can enhance the expression of programmed death-ligand 1 (PD-L1), a pivotal immunological checkpoint, as a mechanism to evade destruction by CD8+ T lymphocytes. A subset of circulating tumor cells during dissemination is responsible for seeding metastases. While research is ongoing, elevated PD-L1 expression in these cells among lung cancer patients is associated with an unfavorable prognosis (116, 117). Similarly, colorectal cancer metastases exhibit higher PD-L1 expression than the original lesions (118).

DTCs expressing PD-L1 may evade elimination by CD8+ T cells, establishing metastatic spread. In the case of pancreatic cancer, tumor cells can disseminate to the liver and remain dormant until conditions favorable for metastatic expansion arise. Notably, adaptive immunity exerts a selective pressure on DTCs that reach the liver, leading to the destruction of the majority of DTCs. However, quiescent cancer cells lacking Major Histocompatibility Complex class I (MHC class I) can evade T cell assault (119). Antigen presentation and CD8+ T cell recognition are contingent on tumor cells expressing MHC class I; therefore, DTCs with insufficient MHC class I evade identification and elimination by CD8+ T lymphocytes. As previously reported, the immune evasion mechanism of latent DTCs is akin to that of quiescent epithelial stem cells. This suggests that metastasis-initiating cells (MICs) may adopt immune evasive abilities similar to those observed in tissue stem cells (120). It has been investigated that elevated infiltration of CD8+ memory T cells restricts the metastatic invasion at earlier stages (121, 122), thus associating the CD8+ memory T cells with better survival in colorectal cancer patients (122). Accordingly, our recent study found that deletion of HIF1α reduces the B16 melanoma tumor growth and expands the population of effector memory function of CD8+ T cells (123, 124). This suggests the positive correlation of HIF1α with the CD8+ T cell exhaustion, exerting oncogenic roles and exacerbating tumor metastasis.

According to reports, CD4+ T lymphocytes with specificity for tumors can create pre-metastatic niches in bone by secreting receptor activators of nuclear factor-κB ligand (RANKL). This secretion, in turn, increases osteoclastogenesis and promotes the metastasis of breast cancer cells to the bone (125). Notably, the conditioned media from 4T1 breast cancer cells induces the expression of chemokine CCL22 in the lung stroma, subsequently elevating Treg levels in the pre-metastatic lung (126). Targeting Treg is an effective anti-cancer therapy as the role of Tregs, particularly the tissue-specific Tregs, in the progression of cancer and cancer metastasis is well studied (127–129). Accordingly, our previous investigation illustrates the elevated intra-tumoral Treg frequency in HIF1α depleted mice. At the same time, combinational therapy using HIF1α inhibitors and Treg inhibitors synergistically boosted anti-tumor immunity and decreased tumor metastasis (123).

The differentiation of naïve CD4+ T cells into Th1 or Th2 cells, which drive inflammatory and anti-inflammatory responses, respectively, plays a role in promoting cancer growth, compounded by the systemic inhibition exerted by tumor-specific Tregs. An imbalance in Th1 and Th2 differentiation has diminished pulmonary immune surveillance (130). For example, oxygen-sensing proteins expressed by T cells can reduce the lung Th1 immune response, which is crucial for maintaining an immunoregulatory state that prevents unnecessary inflammation in response to benign foreign antigens. Conversely, suppressing Th1 immunity in favor of Th2 immunity creates an immune-permissive environment that encourages metastatic colonization (130). Furthermore, the myeloid cell populations in the pre-metastatic niche have been reported to promote the differentiation of T cells into anti-inflammatory Th2 cells (130, 131). This underscores the collaborative efforts of various lymphoid cell populations in establishing a favorable environment that inhibits immune surveillance mediated by T and NK cells, ultimately promoting the spread of metastases. As this is an emerging study area, further investigation is warranted to elucidate the significance of non-myeloid cells and their interactions with myeloid cells in forming pre-metastatic niches.

### Natural killer cells in the metastatic cascade

Natural Killer (NK) cells are crucial in protecting the body against cancer by eliminating cells lacking MHC class I. DTCs must evade NK cell surveillance to survive and spread (132, 133). A balanced signaling mechanism intricately regulates the ability of NK cells to destroy tumor cells. NK inhibitory receptors recognize MHC class I, while activating receptors, such as NKG2D, bind to ligands on tumor cells. NKG2D ligands, like UL16-binding proteins, can undergo altered expression in cancer through processes such as microRNA modulation, transcriptional regulation by factors like IFNγ, DNA methylation, or low histone acetylation (134–136). Additionally, metalloproteinases can cleave NKG2D ligands from the cell surface or secrete them in exosomes, thereby diminishing NK cell assault on cancer cells (136–139).

Quiescent DTCs have been described as downregulating ligands for NK cell activating receptors, providing a mechanism to evade NK cell assault in lung and breast malignancies, particularly those expressing Sox2 and Sox9 and adopting stem cell-like characteristics (140). Moreover, overexpression of Sox9 in metastatic lung tumor cells by upregulating MHC class I is a protective shield against NK destruction (141). Notably, groups of DTCs exhibit more excellent resistance to NK cell-mediated clearance than individual DTCs, possibly due to their ability to suppress ligands activating NK receptors (142). Furthermore, tumor cell clusters may attract additional immunosuppressive myeloid cells in the metastatic niche, potentially thwarting NK cell activity (143, 144). These findings highlight that the regulation of DTCs by NK cells may depend on cancer cell characteristics, including their proliferative status, and may evolve as micro- and macrometastases.

## Non-cellular components involved in the metastatic cascade

### Extracellular matrix in the metastatic cascade

The extracellular matrix (ECM) is responsible for performing various primary activities mediating the fate and function of both normal and tumor cells (145). ECM is generally organized by combining numerous matricellular-associated, fibrous, and proteoglycan proteins (146). ECM is regarded as a dynamic regulatory process that involves a tight regulation of organization, posttranslational modifications, and composition of ECM, which in turn supervise the biochemical and mechanical properties of ECM (147).

In normal physiological circumstances, the stroma cells and parenchymal cells interact with each other by secreting molecular messengers into the microenvironment, by cell-cell contact and communication, or by exerting multiple biophysical and biochemical cues through ECM (148). This interaction results in maintaining tissue homeostasis and establishing niches that bear the characteristics of a distinct microenvironment to ease the growth and survival of specialized cells, including stem cells (149). However, under diseased conditions, normal tissue homeostasis is disrupted, which leads to an interruption of the established niches. Generally, disease conditions lead to the development of tumor-causing genetic lesions from the transition of parenchymal cells, which results in the remodeling of the tissue microenvironment, including the cellular components and ECM (150). These tissue microenvironment modifications strongly impact the disease’s pathogenesis and progression (150).

Tumor progression, including the invasive and metastatic properties of tumors, is highly associated with EMT. Procuring a mesenchymal phenotype is highly dependent on the increased motility of tumor cells, enhanced survival of tumor cells, and elevated expression of enzymes involved in ECM remodeling, such as MMPs. All these changes are necessary for tumor cells to cross the basement membrane, endorse their interaction with ECM, and finally, the intravasation and longevity within vessels (151). Meanwhile, activation and expansion of fibroblasts, elevation in crosslinking and remodeling of ECM, enhanced angiogenesis, and chronic inflammation lead to the stiffness of tissue stroma and tumor desmoplasia (152). Furthermore, tissue stiffness and tumor desmoplasia occur when the tissue exhibits abnormal homeostasis and elevated levels of ECM proteins, such as type I collagen, matricellular proteins, proteoglycans, hyaluronic acid, tenascin, and fibronectin (153). Excessive ECM production indicates ECM stiffness and tumor progression (154). It is evident that a highly aggressive cell line of breast cancer, compared to a less aggressive breast cancer cell line, exhibited increased collagen content and cross-linking, which ultimately enhanced the ECM stiffness (155). During tumor progression, the stiffness and cross-linking of ECM are tightly regulated by TGFβ, which exerts substantial influence on the adjacent fibroblasts and other stromal cells. A myofibroblast phenotype in prostate cancers showed increased levels of smooth muscle α-actin, tenascin, vimentin, and collagen I, along with an increase in TGF-β1 within prostatic intraepithelial neoplasia (156). TGF-β1, therefore, encourages the stromal fibroblasts’ contraction and consequent ECM stiffness, which is linked with tumor aggressiveness (157, 158). Conclusively, ECM stiffness induces TGF-β-dependent EMT and stimulates a basal-like cancer cell phenotype which encourages the metastatic tumor [91], whereas reducing the ECM stiffness and crosslinking alleviates tumor metastasis (159, 160). Similarly, tumor ECM is also known to increase tumor cell motility (161). It was evident that TGFβ stimulates the CAFs in the colon, which secretes various factors, such as tenascin C and hepatocyte growth factor (HGF), to play influential roles in the invasion of colon cancer cells (162). In contrast, fibroblast-derived ECM in the TME directs cancer cells to stick to the cancerous matrices and participates in the metastatic cascade (163).

Tumor cells display few sense transmembrane receptors, e.g., syndecans, discoidin domain receptors (DDRs), and integrins, to sense the ECM mechanical and biochemical properties (164). Activation of these receptors by recognizing the specific motifs within the ECM activates downstream signaling pathways to impact the tumor cell behavior (164). Elevation in stiffened ECM and the crosstalk between integrins and other receptors can activate the Rho GTPase activity, aiding in tumor cell migration. Briefly, stimulation of RhoA GTPase activates phosphorylation of ROCK which ultimately induces the contraction of actomyosin (165). Meanwhile, activation of Rho GTPase through integrins is involved transcription of multiple genes which are vital in tumor growth, motility, differentiation and survival (165, 166). Besides that, collagen also induces the integrins which initiate a cascade of events comprising the recruitment and activation of molecules involved in focal adhesion, such as Talin, FAK, Rho and Ras. These focal adhesion molecules promote the progression of tumors, and lead to the contraction of tumors cells (167, 168). Increased cross-linking and stiffness of ECM also promotes the FAK phosphorylation, and enhances the cancer progression; however, decrease in tissue tension and ECM stiffens minimizes the FAK activity and lowers the invasion and metastasis of tumor cells (160, 169). Similarly, it was studied that ECM stiffness also activates the PI3K signaling which is involved in various cellular processes, including metastasis (170, 171).

**Figure 2:**
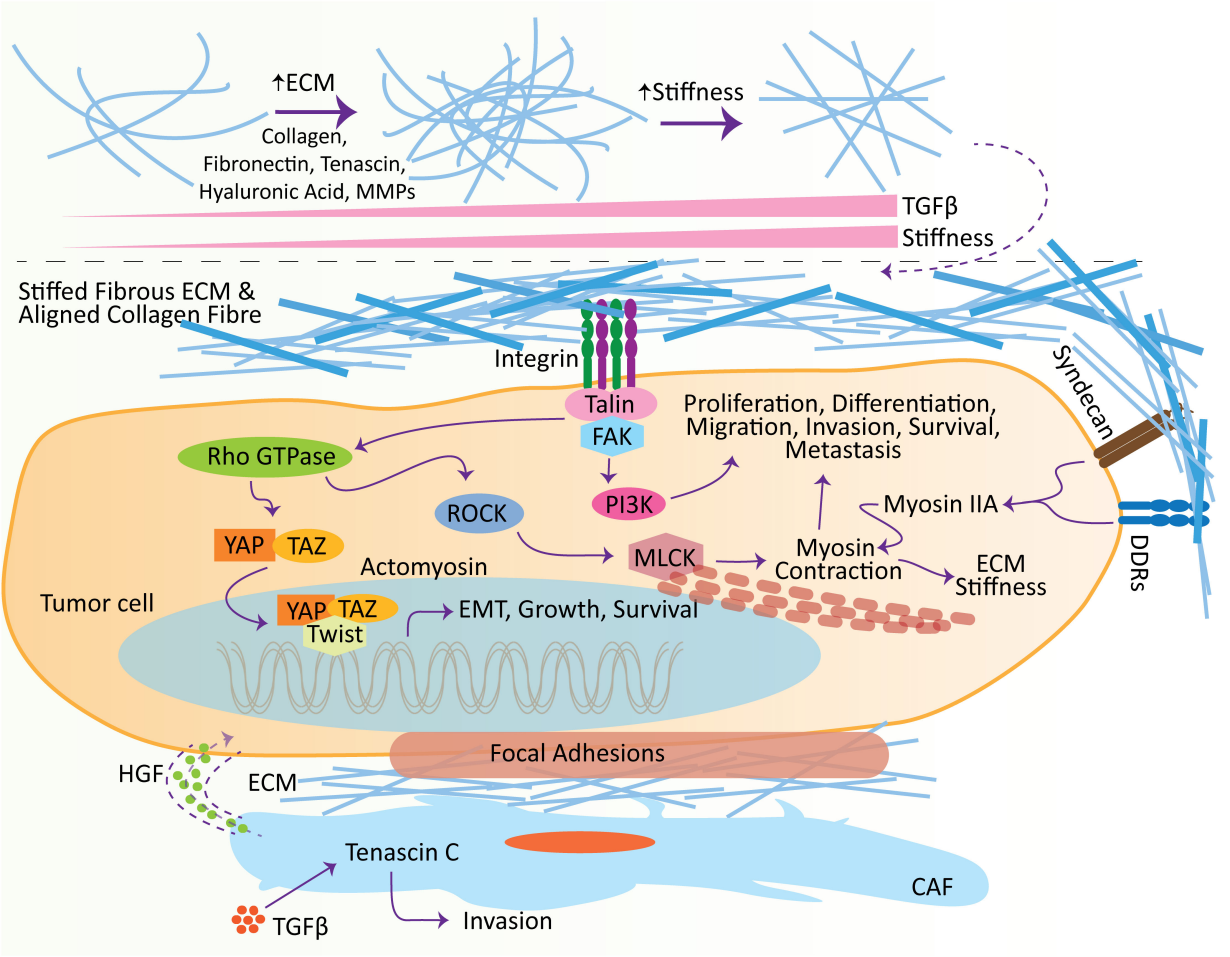
Effect of extracellular matrix (ECM) in promoting metastasis. ECM influences several hallmarks of cancer at different stages, including initiation and metastasis. With the increase in TGFβ, ECM molecules undergo remodeling, which leads to the stiffness of the ECM. Stiffens ECM binds to receptors, e.g., syndecans, DDRs, and integrins, located on the surface of tumor cells to induce intracellular pathways. Crosstalk between ECM ligands and integrins potentiates the Talin, which subsequently activates the focal adhesion kinase (FAK) to induce the adhesion complexes’ assembly, e.g., PI3K-Akt signaling pathway, which plays vital roles in the tumor cell proliferation, differentiation, migration, invasion, survival, and metastasis. Meanwhile, the crosstalk of integrin and growth factor receptors also activates the Rho GTPase. Rho GTPase consequently induces the ROCK, elevating the phosphorylation of MLCK and promoting actomyosin contraction. Furthermore, ECM ligand interaction with DDRs and syndecans induces cell migration and contraction by shortlisting numerous molecules, including myosin IIA. Additionally, ECM stiffness promotes the adhesion signaling integrin clustering and translocates Twist1 and YAP into the nucleus to induce EMT. Similarly, TGFβ encourages the level of Tenascin C in the CAFs, which is involved in cell invasion. These CAFs excrete HGFs to establish the niches in tumor cells. CAF, cancer-associated fibroblasts; TGFβ, transforming growth factor-β; ECM, extracellular matrix; HGF, hepatocyte growth factor; DDR, discoidin domain receptor; MMP, matrix metalloproteinase; FAK, focal adhesion kinase.

The primary effectors of Hippo signaling pathways YAP and TAZ are known to be involved in regulating several cellular and biological processes, including cell proliferation, tumor metastasis, tissue homeostasis, cell differentiation, immune regulation, and tumor microenvironment (172, 173). During the activation of the Hippo signaling pathway, transmembrane cadherin FAT recruits the cascade of Ser/Thr kinase, which phosphorylates the YAP/TAZ and establishes a 14-3-3 binding site. Binding for 14-3-3 with YAP/TAZ hinders the transcription of YAP/TAZ target genes by confiscating this complex in the cytoplasm and hindering its translocation in the nucleus (174). Interestingly, integrins-mediated Rho GTPase-ROCK regulates the YAP in Hippo pathway-independent manner (175, 176), thus associating the gene transcription and mechanotransduction with promoting cell growth (177). Moreover, matrix stiffness induces the integrin cluster and Twist1 detachment from G3BP1 in the cytosol. This translocates the Twist1 into the nucleus, where it performs a critical role in driving EMT (178). Meanwhile, ECM stiffness influences the YAP phosphorylation and nuclear localization to affect cell growth (179). Collectively, ECM, collagen cross-linking and stiffness modulate the numerous keys signaling pathways involved in cell growth, differentiation, migration, invasion, survival, and metastasis (148, 164) (**Figure 2**).

### Hypoxia in the metastatic cascade

Oxygen, an essential factor obligatory for oxidative metabolism and adenosine 5’-triphosphate production, maintains normal tissue homeostasis and developmental processes in cells. The metazoans are using the hypoxic signaling pathway to regulate oxygen homeostasis (180). Hypoxia is an oxygen-deprived condition regarded as a crucial factor in TME. Hypoxia regulates numerous hallmarks of cancer, such as immune evasion, EMT, invasion, angiogenesis, stemness, and metastasis (3, 181). During the hypoxic conditions, hypoxia-inducible factors (HIFs), HIF-1 and HIF-2, activate numerous genes involved in cell apoptosis, differentiation, proliferation, angiogenesis, erythropoiesis, metabolism, and glucose uptake (180, 182). Increasing evidence denoted that tumor initiation, progression, and metastasis are positively correlated with HIF signaling (183).

It was assessed that impaired oxygen homeostasis, in terms of an imbalance between oxygen delivery and oxygen consumption, can cause regions of hypoxia and/or anoxia in 50 to 60% of solid tumors (184). In the settings of TME, abnormal tumor vasculature comprising the distended and leaky capillaries leads to an impaired oxygen supply (185). Meanwhile, the overwhelming population of cancerous cells and infiltrating immune cells increases the oxygen consumption (186). It was verified that oxygen-deprived cells show more aggressiveness and invasiveness with enhanced metastasizing ability. For example, inoculation of hypoxia-induced multiple myeloma cancer cells in mice displayed a faster dissemination rate to the bone marrow than normoxic cells (187, 188). Similarly, an elevated amount of lymph node metastases was observed in the cervical carcinoma disease model, which was exposed to acute hypoxia (189). Likewise, acute hypoxia-induced lung metastases in sarcoma tumor-bearing mice (190). Furthermore, clinical evidence also advocates that hypoxia is highly related with resistance to metastasis, radiotherapy and chemotherapy, poor patient survival, and the activation of HIF pathways (191–193).

EMT is a well-known physiological process active during embryogenesis and tissue regeneration (194). Besides, EMT also plays a critical role in the progression and development of tumorogenesis, especially in hematologic malignancies (188) and numerous solid tumors (195). It was noted that TGF-β and hypoxia act as a master regulator to promote EMT, which further activates numerous downstream transcriptional factors, including Twist, Slug, TCF3, Snail, ZEB1, and Smads (188, 196, 197). The underlying mechanism for hypoxia significantly impacted the EMT, which in turn changes the expression profile of multiple genes and affects the invasive and migratory capability of tumor cells (198). In brief, hypoxia-induced EMT decreases the expression of genes involved in epithelial regulation, for instance, β-catenin and E-cad (199), and elevates the expression of genes involved in mesenchymal-regulation, for instance, CXCR4 (187, 188), SMA, vimentin (200) and N-cad (201).

Under the influence of an oxygen-deprived microenvironment, hypoxia-inducible factors (HIFs) primarily regulate the expression of genes. Generally, HIFα and HIFβ subunits of the heterodimeric HIF complex mediate the HIF-dependent transcriptional regulation by binding to the hypoxia-responsive element (HRE) located within the promoter of target genes (202). It was found that the inactivation of HIF1α and HIF2α selectively revokes metastasis without affecting the formation of primary melanoma, suggesting the prime role of HIF signaling in the development of metastatic tumors and shaping the metastatic niches (203). Similarly, hypoxia and HIF signaling regulate lysyl oxidase (LOX) levels and ECM protein. It was noted that the upregulation of LOX is associated with distant metastasis and increased invasiveness of hypoxic cancer cells through cell-to-matrix adhesion and focal adhesion kinase (204). Meanwhile, LOX, secreted by primary hypoxic tumors, plays an essential role in forming a premetastatic niche by crosslinking with collagen IV and recruiting CD11b^+^ myeloid cells (205). Carbonic anhydrase IX (CAIX) is a downstream gene of HIF1α signaling, induced under hypoxic conditions and is widely available at the tumor site. It was investigated that CAIX induces the invasion and survival of tumor cells by regulating the intracellular and extracellular pH (206). Furthermore, CAIX was also associated with reduced metastasis and tumor volume in tumor models of breast cancer (206). Additionally, C-X-C chemokine receptor type 4 (CXCR4) is activated in tumor cells under oxygen-deprived conditions. This CXCR4 performs a crucial function in cell trafficking (207, 208). SDF-1 acts as a ligand for the CXCR4. Recent studies exhibited that metastatic malignant cells that express an elevated level of CXCR4 are also enriched with SDF-1, and targeting the CXCR4/SDF-1 axis can significantly disrupt the metastatic cascade (187, 209).

A new term, intermittent or cyclic hypoxia, is registered in multiple studies that temporarily describe the shutting down of inefficient vasculature in tumors. This could result in hypoxic and reoxygenation periodic cycles within cancer cells (210, 211). During intermittent hypoxia and hypoxia-induced EMT, HIF1α serves a central role in enhancing the aggressiveness of tumor cells by regulating numerous genes involved in EMT (212, 213). In the tumor, endothelial and hepatic cells, TGFβ and HIF1α activate each other (214–217). Moreover, HIF1α promotes breast cancer progression by modulating the TGFβ1/SMAD3 axis (218). Also, a study conducted using pancreatic cancer cells indicates that HIF-1α regulates the EMT and invasion of cancer cells through hedgehog signaling (219). Furthermore, HIF1α also drives the EMT in hepatocellular and prostate carcinoma through Wnt/β-catenin signaling (220, 221). Interestingly, in prostate cancer and pancreatic cancer, HIF1α exerts its physiological role on EMT indirectly through FoxM1 signaling (222) and the PAFAH1B2 gene (223), respectively. Similarly, HIF1α interacts with integrin-linked kinase (ILK) by establishing a mediating loop and inducing ILK expression to enhance EMT in prostate and breast cancer cells (224).

**Figure 3:**
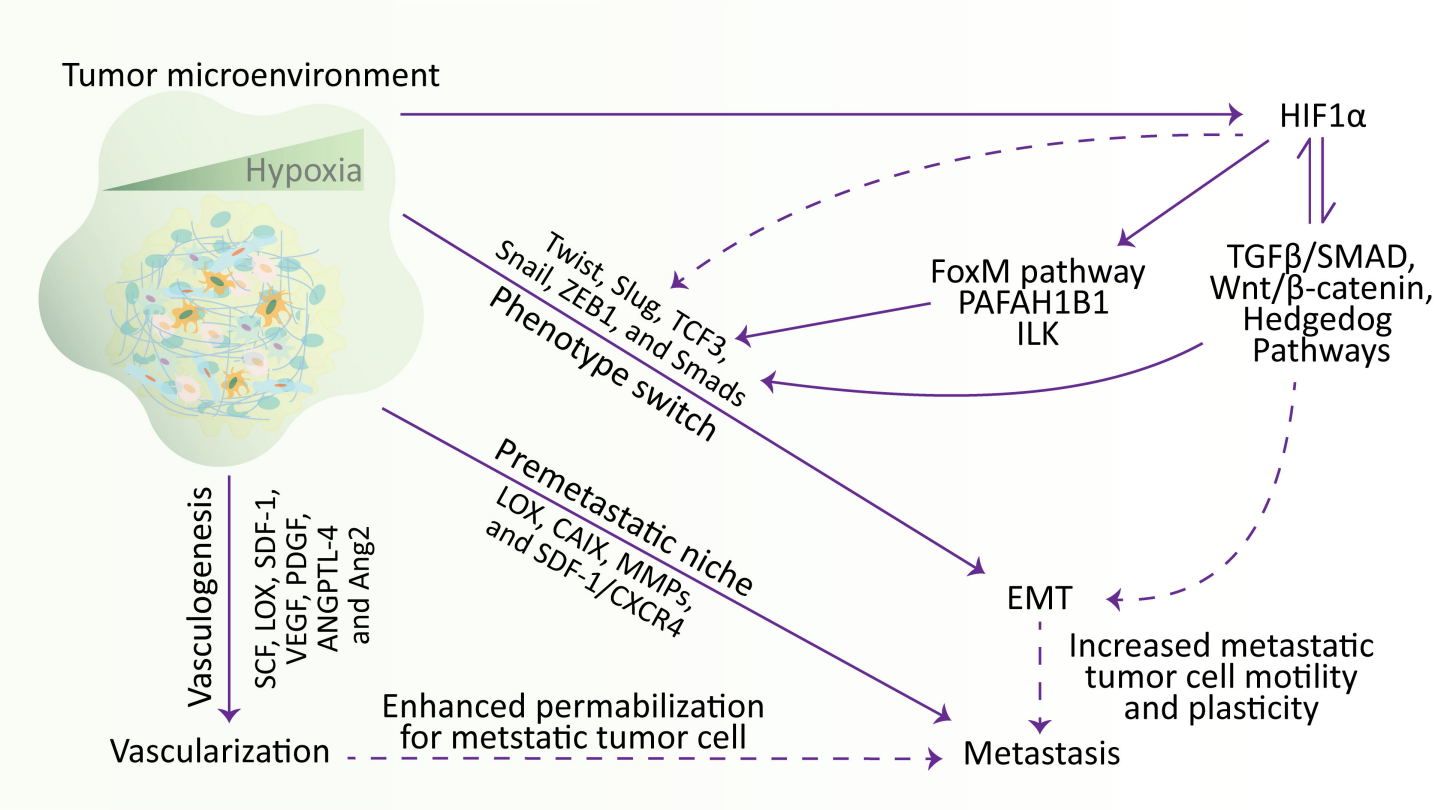
Hypoxia regulates the metastatic cascade. In the TME, hypoxia stimulates EMT, metastasis, and vascularization. Briefly, hypoxia stimulates the vascularization via influencing SCF, LOX, SDF-1, VEGF, PDGF, ANGPTL-4, and Ang2 genes, participates in metastasis by establishing a metastatic niche and modulating the expression of LOX, CAIX, MMPs, and SDF-1/CXCR4, and increase the expression of Twist, Slug, TCF3, Snail, ZEB1, and Smads transcription factors to affect the mobility and plasticity of tumor cells to undergo EMT. Meanwhile, hypoxia activates the HIF1α, which induces the transcription factors involved in EMT through various signaling pathways. Additionally, hypoxia-regulated EMT and vascularization also participate in metastasis by increasing cell motility and plasticity and enhancing permeabilization for metastatic tumor cells, respectively. EMT, epithelial-to-mesenchymal transition; HIF, hypoxia-inducible factor.

Another important function of HIF1α is that it is involved in mediating the expression of multiple immunosuppressive molecules, which are associated with the cancer cells’ development, progression, and metastasis, and possibly ignite the EMT in a TGF-β-dependent manner (225, 226). Induction of EMT retaliates by provoking numerous immune-regulatory effects, including the apoptosis of natural killer (NK) and T-cells but an increase in B cells and Tregs population (227). Similarly, as mentioned earlier, stromal cells regulate cell proliferation, adhesion, and survival by easing the development and dissemination of tumors. Hypoxia affects the production of multiple factors, such as SCF, LOX, SDF-1, VEGF, PDGF, ANGPTL-4, and Ang2, by inducing stromal cells. These factors influence lymphangiogenesis and the formation of new blood vessels. Meanwhile, SDF-1, derived from stromal cells, entices the tumor cells to facilitate metastasis (228, 229). Together, these studies indicate that HIF1α endorses numerous transcriptional factors and modulates various signaling pathways to play a central regulatory role in the hypoxia-induced EMT and metastatic cascade (**Figure 3**).

### Gut microbiota in metastatic cascade

Host microbes are crucial regulators in modulating the susceptibility to the development and progression of tumors. These microbes employ their functions markedly through various indirect mechanisms, including their metabolites and influencing the immune system at proximal or distant tumor sites (230, 231). Various reports have indicated that tumor-resident microbes are highly associated with the increased risk of cancer progression (232) and cancer metastasis (233). Meanwhile, microbiota also influences the efficiency of immunotherapy and chemotherapy (234). Gastrointestinal tumors normally metastasize to the lungs, liver, and regional lymph nodes. In this process, gut-microbiota initiates metastatic cascade and promotes tumor growth with restricted response to anti-tumor therapy (235). How microbiota initiates the metastatic cascade has not yet been cleared. An investigation urged that the bacteria may exert a crucial role in the TME as they can infect the tumor cells at the primary site. Infection of tumor cells with bacteria is maintained till the colonization of tumor cells at distant metastatic sites, indicating the stability of the microbiome throughout the progression of the tumor from primary site to the metastatic site (236). In compliance with this, a recent study determined that numerous intracellular bacteria can undergo circulation with tumor cells during the metastatic cascade and play critical roles in metastatic colonization. Briefly, the researchers found that tumor-intracellular microbes modulate the actin cytoskeleton in the host cells and promote the overall survival and viability of metastatic tumor cells against the fluid shear and tear-out stress during the process of circulation (237).

The gut microenvironment, particularly in disease conditions, is enormously complex and comprised of countless bacteria and their products/metabolites (238). Sudden changes in the gut microflora considerably impact the colorectal cancer (CRC) microenvironment, which may also influence the progression and recurrence of colon cancer. Notably, reduced *Bacteroides* and enhanced *Clostridial* numbers may improve CRC-liver metastasis (239). Genetic ablation of p53, a tumor suppressor, induces colon cancer, promotes its progression, and increases intestinal permeability. This helps in the development of an inflammatory microenvironment in an NF-κB-dependent manner, which enhances the EMT and dissemination of metastatic tumor cells to the lymph nodes (240). In normal circumstances, gut microbiota has the ability to reduce the expression of p53 through post-translational modifications, transcriptional inhibition, and protein degradation (241).

Interestingly, the majority of the metastatic tumors in CRC models are observed in the liver. This is due to the continuous exposure of the liver to the gut microbial products/metabolites through enterohepatic circulation and portal vein, which extends the direct connection between the gut and liver, particularly during liver metastasis (242, 243). Gut microbiota establishes an immune suppressive hepatic microenvironment by regulating the inflammatory cytokines, such as IFN-γ, TNF-α, IL17, IL12 and IL6, which facilitate the development of hepatic metastatic tumor (244, 245). In parallel, gut microbiota influence the bile acid metabolism to regulate the population of intrahepatic natural killer T (NKT) cells through the regulation of CXCR16 in hepatic sinusoidal cells, which ultimately affects hepatic metastasis (244).

Kynurenine (Kyn) emerges as the primary product in the tryptophan metabolic pathway, regulated by IDO and TDO2 in tumor cells. Kyn has been shown to activate the AhR, initiating autocrine and paracrine functions that impact the anti-tumor immune response and tumor cell survival and migration (246). Its involvement in the induction of Slug expression inhibits E-cadherin, a crucial regulator of cell adhesion. Additionally, the AhR ligand, dibenzo-p-dioxin (TCDD), has been found to enhance the expression and function of MMP9 across various cancers, including melanoma, urothelial, prostate, and gastric cancer cells (247). AhR has also promoted EMT induced by polychlorinated biphenyls in HCC cells (247, 248). In the context of renal cell carcinoma, the study investigated the impact of AhR on EMT development. The findings revealed elevated AhR expression in RCC through the gut microbiota-derived tryptophan metabolite Kyn promoted the migration and invasion of 786-O cells while inhibiting cell death, suggesting a role for AhR in these processes (249).

In CRC, *Fusobacterium nucleatum* has been studied extensively. *F. nucleatum* elevates the invasion, proliferation, and recurrence of CRC through the stimulation of pro-inflammatory cytokines (250, 251). It was reported that *F. nucleatum* enhances the development of metastatic hepatic tumors in CRC by blocking the activity of NK cells (252), restricting the activity of cytotoxic T cells (253), inducing the pro-inflammatory pathways (254), and prompting the overall mucosa-associated inflammation (255). Remarkably, *F. nucleatum* is translocated to the hepatic metastatic tumor, signifying that *F. nucleatum* may assist the metastatic tumor cell migration, colonization, and proliferation at distant tumor sites during CRC (236).

**Figure 4:**
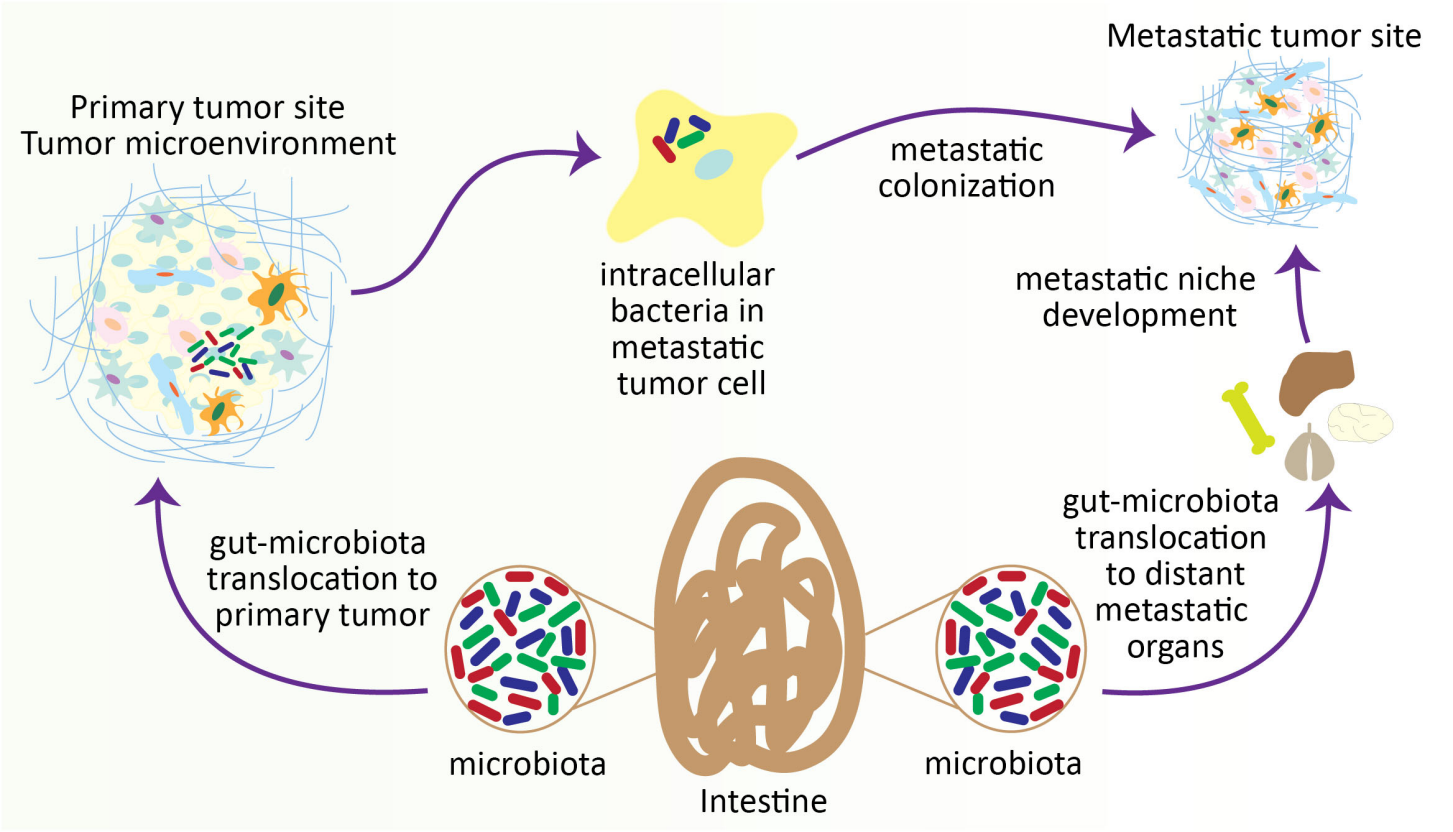
Gut microbiota contributes to colonizing metastatic tumor cells in distant organs. During the progression of tumors, particularly in intestinal tumors, the gut microbiota translocates to the distant metastatic organ and develops a metastatic niche. On the other hand, gut microbiota also penetrates the metastatic tumor cells within the TME and helps the colonization of metastatic tumor cells at the metastatic site.

In addition, *Escherichia coli* also interrupts the enterohepatic vessels to establish the premetastatic niche and endorses the recruitment of APCs for a pro-inflammatory environment suitable for colonizing metastatic tumor cells (256). *E. coli* has been implicated in an elevated risk of colon cancer due to its ability to synthesize colibactin, a genotoxic strain of bacteria. Colibactin induces dysbiosis in the gut microbiota, leading to DNA double-strand breaks and activating the Wnt/β-catenin and NF-κB pathways. This genotoxic effect, coupled with inflammation of the colonic mucosa, creates an environment conducive to cell proliferation, thus promoting the development of colon cancer (257, 258). In addition to E. coli, the gut microbiota harbors Fragilysin, another factor associated with colon cancer. Fragilysin binds to epithelial receptors in the colon, triggering the NF-κB pathway. This activation, in turn, enhances colon cell division, growth, and induces DNA damage (259, 260). Notably, Fragilysin is also implicated in the cleavage of E-cadherin, leading to the deregulation of the Wnt/β-catenin signaling pathway. This process further contributes to cell division and activates c-MYC, emphasizing its role in promoting the development of colon cancer (259–261). These studies prove the role of gut microbiota in the progression of tumors and the development of metastatic tumors at distant sites (**Figure 4**). Meanwhile, specific experiments do not validate how the microbiome influences cancer metastasis. Thus, additional studies are required to investigate the detailed molecular mechanism of the microbiome regulating the pre-metastatic niche development and the migration and colonization of metastatic tumor cells.

### Circadian rhythm in metastatic cascade

**Figure 5:**
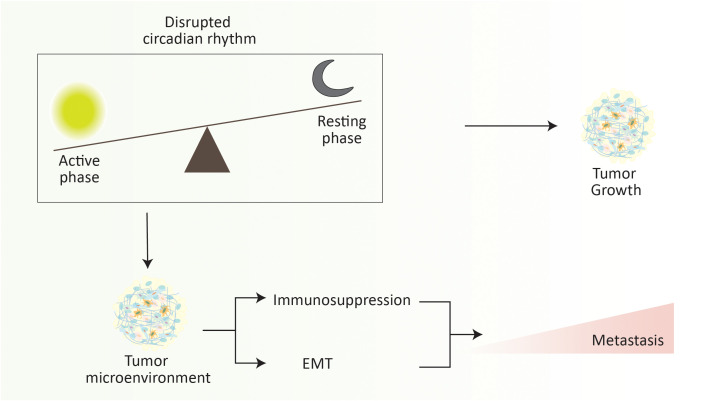
Disruption of circadian rhythm elevates the metastasis progression. Abrupt changes in the active phase and resting phase of an individual significantly increase the metastasis rate. It is noteworthy that the overall rate of metastasis increases during the resting phase. Briefly, disruption of the circadian rhythm promotes the immunosuppressive tumor microenvironment and EMT to elevate the risk of cancer metastasis.

The circadian clock regulating the circadian rhythm is an evolutionarily conserved mechanism that modulates ample molecular, cellular, and physiological processes (262). It is a 24-hour intrinsic clock that helps our brain regulate the time, being active and resting by retorting the changes in environmental light. Recent advances in cancer research have directed the role of circadian rhythm in cancer progression and metastasis by regulating various cellular processes (263, 264). Circadian rhythm plays a critical role in the metastatic cascade by influencing the dissemination, circulation, and intravasation of metastatic tumor cells, which was initially documented by understanding the role of melatonin (a hormone that acts as a time cue) in the tumor progression and metastasis (265–267). For instance, in a spontaneous mouse model of mammary carcinoma, severe disruption of the circadian rhythm elevates the dissemination and metastasis of tumor cells (**Figure 5**). In brief, disruption of circadian rhythm aggravates the stemness and tumor-initiating potential of tumor cells, thus diminishing the antitumor immunity (268). Similarly, disruption of the circadian cycle affects distant lymph node metastasis in a transplantation-arbitrated breast carcinoma without distressing the tumor growth at the primary site (269).

Even though the role of circadian dynamics in the progression of tumor metastasis is poorly understood and has been linked to epidemiological studies (270–272), several experimental studies have discussed the role of circadian rhythm in the metastatic cascade (263, 273). Experimentally, it was validated that intravasation of circulatory tumor cells at distant metastatic sites is highly dependent on regulating the circadian clock, as metastatic breast tumors were increased in both human and animal breast cancer during sleep (273). Similarly, the fluctuation of daily circadian rhythm alters the population of circulatory tumor cells in multiple myeloma (274), and prostate cancer (275). However, the time when these metastatic tumor cells reach the peak is controversial. Bmal1, a circadian clock-related gene, regulates TGF-β expression by targeting PAI-1. It was noted that reduced Baml1 increases TGF-β functions by enhancing the plasmin production, thus leading to tumor metastasis (276).

A recent report has shown that the number of single circulatory tumor cells and the circulatory tumor cell cluster cell exacerbate at the resting phase of circadian rhythm along with an increased rate of intravasation (263). Meanwhile, the rate of metastasis due to heterotypic clusters, such as tumor cells with neutrophils or fibroblasts, increases during the active phase (263). This was previously verified in melanoma cancer, which progressed toward lung metastasis resulting from a circadian pattern. The researchers established that the lungs had more metastatic lesions during the resting phase of the circadian rhythm. Meanwhile, it was also indicated that the accumulation of neutrophils in the lungs aided in developing a metastatic niche in the lung in the resting phase, which supported the localization of metastatic tumor cells (277). In addition, the resting phase also aggravates the development of lung metastasis in a model of breast cancer and also demonstrates the role of circadian rhythm in the development of premetastatic niche and localization of metastatic tumor cell at distant metastatic site (273).

While there is considerable knowledge about circadian rhythms, understanding how they function in various disease states remains limited. Disruption of circadian clocks has been linked to accelerated tumor growth rates and a higher incidence of cancer (278, 279). Recent reports suggest that cancer cells exploit their circadian cycles to promote metastasis through disruption (273). BAML1, an important component of circadian rhythm, has been implicated in stimulating human cancer cell growth, migration, survival, and invasion. The intricate interplay between circadian rhythm and cancer metastasis involves various molecular mechanisms. One such mechanism is the PAI-1-TGF-β-myoCAF-dependent pathway, which promotes metastasis when the clock component BMAL1 is disrupted in mice (276). In colorectal cancer, BMAL1 plays a pivotal role in stimulating the migration and invasion of cancer cells. This effect is mediated through the upregulation of c-Myc expression, a process dependent on the activation of ERK and JNK signaling pathways. The dysregulation of BMAL1 in this context enhances the invasive potential of colorectal cancer cells, contributing to the metastatic cascade (280). Another facet of the circadian regulation of metastasis involves the downregulation of BMAL1 by miR-494-3p in hepatocellular carcinoma. This downregulation results in the upregulation of GPAM-mediated lipid biosynthesis, fostering the growth and metastasis of hepatocellular carcinoma cells (281). Furthermore, in breast cancer, the circadian protein BMAL1 emerges as a regulator of metastasis by influencing the expression of matrix metalloproteinase9 (MMP9). The increased expression of MMP9, orchestrated by BMAL1, enhances the invasive potential of breast cancer cells, contributing to their ability to invade and metastasize (282). Similarly, the downregulation of the circadian rhythm regulator HLF (Hepatic Leukemia Factor) has been identified as a critical factor contributing to the promotion of multiple-organ distant metastases in NSCLC. This phenomenon is mediated through the PPAR/NF-κB signaling pathway (283). Collectively, these findings illuminate the significant role of circadian rhythm, particularly the circadian protein BMAL1, in orchestrating molecular events that impact cancer metastasis. The modulation of key signaling pathways and regulatory networks underscores the complexity of the circadian clock’s influence on the metastatic behavior of various cancer types. The core circadian clock transcription factors (TFs) exhibit dual regulatory roles in influencing the induction of EMT. The expression of EMT-TFs, enhancement of stemness, upregulation of EMT markers, and acquisition of EMT-specific cellular characteristics are intricately linked to the downregulation of PER2 in breast epithelial and cancer cell lines (284). Conversely, another study highlights a contrasting effect, revealing that the downregulation of BMAL1 strengthens the epithelial state of colorectal cancer cells. This downregulation results in reduced cell motility, invasion, and drug resistance, suggesting a suppressive role of BMAL1 in maintaining an epithelial phenotype in colorectal cancer (285). Moreover, disturbances in circadian rhythm leading to lower melatonin levels are associated with EMT in rats. Additionally, higher RSK2 overexpression is linked to increased metastatic dissemination of transplanted breast cancer cells. These observations suggest a potential connection between circadian rhythm disruption, melatonin levels, and RSK2 expression in influencing the induction of EMT and subsequent metastatic processes (286).

As previously known, various genes are involved in the tight regulation of the circadian rhythm (287). Experimental studies have stipulated the role of circadian rhythm in tumor progression and metastasis development through genetic modulation of these genes (288). For instance, genetic ablation of Per1 and Per2 declined the development of hepatic metastatic tumors in mice injected with colon cancer cells. This signifies that hampering the circadian rhythm, particularly in TME, may constrain the colonization of metastatic tumor cells by elevating the tumor-suppressive microenvironment (289).

Therapeutic strategies to treat metastatic cancer: The current therapeutic landscape for metastatic disease employs three primary systemic approaches: chemotherapy, targeted therapy, and immunotherapy, often employed in combination. Targeted therapy, which focuses on drugs targeting tumor-driving oncoproteins, has shown substantial improvements in outcomes across various cancers (290). However, cytotoxic chemotherapy remains a cornerstone in metastatic treatment, particularly for cancer subtypes where targeted options are limited. While targeted therapy has demonstrated success, mutation-specific therapy represents another avenue with the potential for striking but often transient responses to tumors. Unfortunately, these drugs frequently contribute to the growth of tumor subclones carrying drug-resistant mutations or capable of evading specific pathways and secretomes. The ongoing efforts in research utilizing patient biospecimens are swiftly identifying mechanisms of resistance, offering valuable insights and potential avenues for advancements in treatment strategies (290–292). For instance, in NSCLC, where first-generation tyrosine kinase inhibitors (TKIs) like erlotinib and gefitinib improved overall survival in metastatic disease but showed limited efficacy in the adjuvant setting (293, 294). In contrast, the third-generation TKI osimertinib, targeting the drug-resistant EGFRT790M mutant, improved survival rates in both adjuvant and metastatic settings (295–297). This underscores the critical need for the development of more effective drugs targeting subclonal disease-resistance mutations, ideally at an earlier stage in the progression of the disease.

Efforts focused on reducing immunosuppressive cross-talk between immune cells, cancer cells, and other components of the metastatic tumor microenvironment hold significant promise (298, 299). The recent breakthrough of combination multi-kinase inhibitor treatment with ICI in metastatic tumors that were normally immune resistant demonstrates the potential of this strategy (300). Liver metastasis-directed treatments are gaining popularity, encompassing various interventions such as hepatic artery infusion chemotherapy, radiofrequency ablation, and embolization (301). In addition to addressing CRC liver metastasis, recent randomized phase 2 studies have demonstrated promising outcomes in individuals with oligometastatic malignancies. In these cases, consolidative radiation therapy is administered to eliminate residual cancer cells in the tumor bed following the surgical removal of the primary tumor. This combined approach has shown increased overall survival in certain patient populations, showcasing the potential of a multimodal strategy in managing oligometastatic disease (302).

A study found that both epithelial cells and immune cells with malignancies not generally linked with microbial interaction, such as those of the breast, ovary, bone, and brain, had a diverse intratumoral microbiome (303). *Fusobacterium* strains have been found to be present in both primary and metastatic CRC and breast cancer, as well as to cause metastasis (236, 304). Antibiotic therapy to lower Fusobacterium load lowered metastatic load in mouse xenografts (236). The makeup and variety of the gut microbiome have been linked to ICI response in metastatic malignancies (305, 306). The bulk of the human microbiome’s metabolites, processes, and therapeutic targets have yet to be discovered. Microbes that attack certain tissue types or attach to cancer cell receptors can also function as drug-delivery vehicles (307). In summary, the evolving landscape of metastatic disease treatment emphasizes the importance of advancing therapeutic strategies, developing more effective drugs, and addressing resistance mechanisms to improve overall patient outcomes.

## Conclusion

Metastasis is acknowledged as one of the leading causes of death among cancer patients; thus, extra attention is being paid to understanding the detailed molecular mechanisms underlying metastasis. TME is one of the critical modulators of metastasis. TME comprises numerous cell types with altered co-factors that contribute to initiating the metastatic cascade and establishing the metastatic niche, ultimately promoting tumor invasion, colonization, and growth at the secondary site. Among these factors, circadian disruption, hypoxia, gut microbiota, and ECM are the prime contributors to tumor progression and metastasis. However, a few studies illustrate an in-depth mechanism of how these TME factors influence the metastatic cascade and the TME. It should be taken into consideration that the microenvironment at the distant tumor site differs from the microenvironment at the primary site. For instance, a new investigation found that TME in the secondary site in the brain was different from the site of origination in the breast (308, 309). Likewise, it was concluded that the tissue of the primary tumor site, e.g., melanoma, breast, or lung, directs the pattern of TME to be established at the distant tumor site and decides the TME-dependent regulation of metastatic tumor growth (309). Moreover, metastatic-tumor cells further promote metastasis by escorting their primary soil from their original site to the secondary site. However, the ECM composition at the secondary site also exerts moderate effects on the composition and components of the pre-metastatic niche environment (22, 310, 311). Meanwhile, a higher degree of inconsistency has been reported in the deposition and stiffness of ECM within an individual tumor (312). However, it has not been studied why the ECM heterogeneity varies and how it impacts the tumor progression.

Coupling ECM remodeling with ECM stiffness is a leading factor contributing to the rapid progression and development of tumors at secondary sites. Stiffened ECM initiates mechanotransduction signals that induce the excretion of MMPs from stromal and tumor cells (313), improving the reorganization and degradation of ECM (314). Therefore, stiffness and mechanotransduction-associated remodeling of ECM is regarded as a dynamic process in cancer which is crucial for immune evasion, tumor angiogenesis, induction of CAFs, migration, invasion of tumor cells, and cancer metastasis. However, further investigations are necessary to examine the molecular mechanisms underpinning ECM stiffness and remodeling-dependent modulation of the tumor microenvironment, tumor metastasis, and immune surveillance.

Conversely, hypoxia exerts a critical role in the progression of cancer metastasis. It is evident that both earlier and later stages of metastasis are being affected by hypoxia in the TME (186). Meanwhile, hypoxia also influences immune regulation by regulating the immune escaping through the promotion of tumor resistance and immune suppression (315). At the primary tumor site, HIF-mediated signaling regulates angiogenesis, EMT, invasion, and migration to facilitate tumor metastasis. Numerous targets of HIF signaling that mediate the invasion and metastasis can serve as potential targets to restrict the growth and development of metastatic diseases. Interestingly, it has been found that hypoxia and circadian disruption also cooperate with each other to enhance invasion and metastasis (316). Furthermore, gut microbiota act as a major influencer on hypoxia and circadian disruption (317, 318). In summary, this review highlights the role of cellular and non-cellular factors, beyond cytokines, influencing cancer metastasis. In the meantime, various advanced investigations have highlighted the divergent mechanisms through which gut microbiota, circadian disruption, ECM and hypoxia influence the progression of metastatic tumors at secondary sites. It is therefore important to identify the new molecular mechanism, therapeutic targets and biomarkers that could assist in diagnosing and treating metastatic disease.

## Author contributions

FR: Data curation, Writing – original draft, Writing – review & editing. JZ: Writing – review & editing. FP: Funding acquisition, Supervision, Writing – review & editing.
